# Evidence of in Utero Anti-*Neospora caninum* Antibody Production in Paired Sow and Umbilical Cord Blood Samples

**DOI:** 10.3390/microorganisms14020477

**Published:** 2026-02-15

**Authors:** Labrini V. Athanasiou, Eleni G. Katsogiannou, Constantina N. Tsokana, Dimitrios Gougoulis, Stavros M. Papadakis, Vasileios G. Papatsiros

**Affiliations:** 1 Clinic of Medicine, Faculty of Veterinary Medicine, School of Health Sciences, University of Thessaly, 43100 Karditsa, Greece; elkatsog@uth.gr (E.G.K.); dgoug@vet.uth.gr (D.G.); papasta68@gmail.com (S.M.P.); vpapatsiros@vet.uth.gr (V.G.P.); 2 Laboratory of Parasitology and Parasitic Diseases, School of Veterinary Medicine, Faculty of Health Sciences, Aristotle University of Thessaloniki, 54124 Thessaloniki, Greece; ctsokana@vet.auth.gr

**Keywords:** *Neospora* spp., swine, umbilical cord, serum, abortion, IgG, IgM

## Abstract

Neosporosis, caused by *Neospora caninum*, is a major protozoal disease responsible for reproductive disorders and economic losses in livestock. Swine are susceptible to *N. caninum* infection, as evidenced by serological and experimental studies, but the impact of natural infection on reproduction failure remains poorly defined. The objective of this study was to investigate *N. caninum* transplacental transmission in naturally infected sows by detecting an active fetal immune response in their stillborn piglets. Paired maternal blood and umbilical cord blood (UCB) samples were collected from 247 sows and stillborn piglets across 39 farrow-to-finish farms in mainland Greece. Sera were tested for anti-*N. caninum* IgG and IgM antibodies using an indirect fluorescence antibody test. An IgG and IgM seropositivity for *N. caninum* of 8.91% and 3.64%, respectively, was reported in sows, while lower percentages of IgG and IgM antibodies (3.24% and 0.81%, respectively) were detected in UCB samples. Overall, antibodies were detected in 4.05% of UCB samples, indicative of in utero antibody production. Positive samples were more frequently encountered on smaller farms with up to 250 sows, possibly due to lower biosecurity standards. The detection of antibodies in UCB resulting from the fetal immune response to intrauterine *N. caninum* infection is indicative of the potential involvement of *N. caninum* parasitism in reproductive system disorders. Testing of UCB for the presence of anti-*Neospora* antibodies elucidates the dynamics of parasite transmission within the farm and provides evidence for the implementation of more efficient biosecurity and preventative measures.

## 1. Introduction

Neosporosis is a parasitic disease with worldwide distribution. *Neospora* spp. is an intracellular parasite of the phylum *Apicomplexa*, class *Conoidasida*, and subclass *Coccidia*, affecting both domestic and wild animals [1]. Antibodies against the parasite have also been detected in humans; however, its zoonotic potential requires further supporting evidence [2].

Among *Neospora* species, *N. hughesi* and *N. caninum* are the main causative agents of the disease. Protozoal encephalomyelitis in horses has been attributed to the former species [3], while experimental infection in mice, gerbils, and dogs has also been reported [4]. Carnivores, including dogs [5], coyotes [6], dingoes [7] and gray wolves [8], are infected by the latter species, *N. caninum*, acting as definitive hosts and shedding oocysts in feces. Ruminants [9,10] and other warm-blooded animals such as pigs [11], deer [12], mice [13], wild rabbits [14], European hare [15], and wild boar [16] serve as intermediate hosts.

The prevalence of antibodies against *N. caninum* varies in different animal species in Greece; in descending order, 20.89% were prevalent in cattle [10], followed by sheep and goats, then in the European hare [15] and wild boar [16] (0.95% and 1.1%, respectively), and the lowest detected prevalence in swine (0.95%) [17]. An intermediate seroprevalence has been reported in dogs ranging from 2.5 to 7.3% [9,18,19].

Worldwide, the prevalence of the detected antibodies against *N. caninum* in swine varies from 1.9% to 27.81% in different countries, including Italy, Egypt, India, China, and Brazil [11,20,21,22,23,24,25]. Moreover, anti-*Neospora* antibodies were found in 15.8% of wild boars from the United States [26], 33.3% of wild boars from Turkey [27], and 18.1% of wild boars from the Czech Republic [28]. A higher seroprevalence has been reported in sows compared to fattening pigs [23]. In addition, the seroprevalence of *N. caninum* in female (17%) swine is reported to be higher than in males (7%), although no association between sex and seroprevalence was found. [29]. Previous meta-analyses on global *N. caninum* seroprevalence in other animal species, such as sheep [30] and cats [31], have shown that both males and females are at equal risk of exposure to *N. caninum* sources of infection.

The coccidian can be vertically transmitted from the mother to the embryo. This can result from either an exogenous transmission of an oocyst-derived infection or reactivation and endogenous transplacental transmission of a latent infection during pregnancy [32,33]. Transplacental transmission of reactivated *N. caninum* or oocyst-derived tachyzoites can lead to reproductive disturbances, including abortions, mummification, stillbirth, and fetal abnormalities in livestock [33]. Besides pregnancy, immunosuppression may also result in reactivation of bradyzoite cysts, rapid replication as tachyzoites, and blood dissemination, causing location-dependent clinical disease. Immunocompromised hosts are more susceptible to developing severe infections with opportunistic pathogens like *N. caninum,* while immunocompetent individuals remain mostly asymptomatic [34].

Transplacental transmission of *Neospora* spp. has been reported in dogs, the definitive hosts, as well as in ruminants [35], with neosporosis being the primary cause of abortions in bovine [36]. That fact makes *N. caninum* a responsible factor for huge economic losses. Domestic swine are suitable intermediate hosts of *N. caninum* [29]. In swine, the first indication of vertical transmission was described in 1998 in experimentally infected swine with *N. caninum* tachyzoites [37]. Recently, transplacental transmission was experimentally verified in all phases of gestation, reporting related reproductive problems [38,39].

A study evidenced an influence of *N. caninum* seropositivity on reproductive parameters in sows, i.e., age at first farrowing, annual number of deliveries, and stillbirth incidence [40]. Recently, Snak et al. [38] demonstrated that in experimentally infected pigs, the parasite could be transplacentally transmitted in all phases of gestation, regardless of the time of infection, causing reproductive disorders and abortion with mummified fetuses, especially in the first- and second-thirds of the gestational period. In addition, due to reactivation of the infection, the endogenous vertical transmission was evidenced in the sows inoculated in the final third of gestation. Moreover, *N. caninum* can cause clinical signs in infected female pigs, including hypothermia and leukocytosis in the acute phase of infection; the infection can also acutely reappear in chronically infected swine during pregnancy [39].

The aim of this study was to assess the percentage of sows and umbilical cords (UCB) of stillborn piglets detected with IgG and IgM antibodies against *N. caninum*.

## 2. Materials and Methods

The study was conducted in compliance with the ethical standards in the Helsinki Declaration of 1975, as revised in 2000, as well as the national law. The study design and all experimental procedures had been approved by our Institutional Animal Use Ethics Committee (Animal Use Ethics Committee of Veterinary Faculty University of Thessaly approval code: 65/26-02-2019 and approval date: February 2019), as this experiment was included in the same project as the one already published [17].

### 2.1. Farm Selection and Samplings

A total of 39 commercial farrow-to-finish pig farms in Greece were included in the study, representing a total population of approximately 10,800 sows (Table 1). The inclusion criteria of farms, based on a questionnaire that was filled in by the farmer owners under provisions of Regulation (EU) 2016/679 as follows:

• The minimum number of breeding stock was 50 sows.
• Vaccination program of breeding stock included at least vaccinations against Aujeszky’s disease virus, parvovirus, *Erysipelothrix rhusiopathiae*, atrophic rhinitis, *Escherichia coli*, *Clostridia* spp. and Porcine Reproductive and Respiratory Virus (PRRSV).
• Vaccination program of weaners included at least vaccinations against *Mycoplasma hyopneumoniae* (*M. hyo*) and Porcine circovirus type 2 (PCV2).
• Preventive program against endo/ectoparasites in breeding stock, using intramuscular administration of ivermectin in sows before farrowing
• Implementation of minimum standards for the animal welfare protection of pigs (Council Directive 2008/120/EC of 18 December 2008).
• A complete and balanced diet is essential for health and welfare according to the nutrient requirements of the National Research Council [41].
• Routine use of anti-mycotoxin agents in the feed of pregnant and lactating sows, as well as those of the weaners.

**Figure 1:**
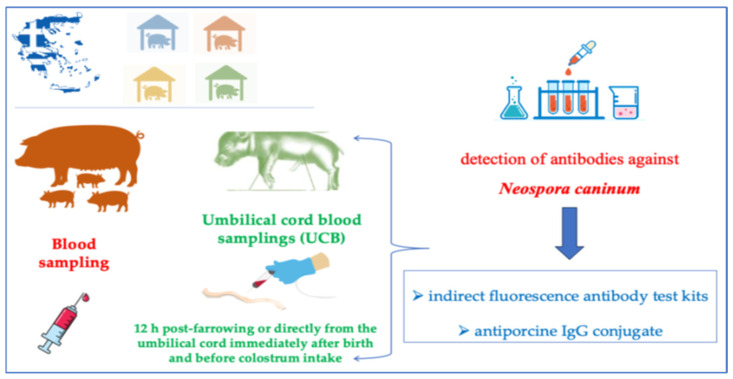
Experimental design of the study.

A total of 247 blood samples were collected exclusively from sows that were actively farrowing at the time of the farm visits and for which the litter included at least one stillborn piglet (type II) (Figure 1). For each of these sows, a maternal blood sample was obtained by puncturing the vena jugularis externa with a 16-gauge needle into a vacutainer (Venoject, Terumo Europe, Leuven, Belgium) without anticoagulant for subsequent serum retrieval. A total of 247 corresponding umbilical cord blood (UCB) samples were collected from the stillborn piglets of the same litter, classified as type II (intra-partum deaths) according to Rangstrup-Christensen et al. [42]. UCB sampling was performed either directly from the umbilical cord immediately after birth and before colostrum intake, or within 12 h post-farrowing. Τhe blood samples were placed in a cooler with icepacks, avoiding direct contact with the tubes, and transferred to the Diagnostic Laboratory, School of Veterinary Medicine, Faculty of Health Sciences, University of Thessaly, Greece. The blood samples were centrifuged at 300× *g* for 10 min, and the sera were transferred to Eppendorf tubes and frozen immediately at −20 °C. The serum was used for the detection of antibodies against *N. caninum*. 

### 2.2. Serological Examination

For the detection of antibodies against *N. caninum*, indirect fluorescence antibody test kits using commercially available slides coated with parasite tachyzoites (MegaFluo®Neospora caninum, Diagnostik MegaCor, Hörbranz, Austria) and antiporcine IgG conjugate (FITC anti-pig IgG conjugate Diagnostik MegaCor, Hörbranz, Austria) were used. For the detection of antibody titers, 1:160 dilution was used as a cut-off. Fluorescence covering the entire periphery of the tachyzoite was considered a positive result [43].

### 2.3. Statistical Analysis

To compare the proportion of seropositive animals between farms, a univariate analysis was performed. Farm size was tested for its association with IgG-positive, IgM-positive, and combined IgG/IgM-positive serological status in sows and umbilical cord blood samples using the χ^2^ test. Fisher’s exact test was applied whenever more than 20% of the expected cell frequencies were below 5. The analyses were performed using MedCalc Statistical Software version 14.8.1 (MedCalc Software bvba, Ostend, Belgium; http://www.medcalc.org; 2014) and the online software(version 2016 Navendu Vasavada) “Fisher’s Test for Exact Count Data” [https://astatsa.com/FisherTest(accessed on 15 July 2024)]. Power (88.2%) was calculated using the total number of paired samples (*n* = 247), the observed IgG seroprevalence (≈9%), and α = 0.05 for detecting an effect size corresponding to an inter-group difference of ~0.08–0.10. The study’s power was calculated at 88.2%, with a significance level of *α* = 0.05.

## 3. Results

Out of the 247 serum samples and UCB samples tested for antibodies against *N. caninum*, those positive for IgG antibodies were 22 (8.91%) and 8 (3.24%), respectively. Positive results for IgM antibodies were found in 9 (3.64%) serum and 2 (0.81%) UCB samples, while positive samples against both IgG and IgM antibodies were 2 (0.81%) and 1 (0.40%), respectively (Table 2).

Despite variations in seropositivity between the different farm size categories, no statistically significant association was found between farm size and the presence of IgG, IgM, or both IgG/IgM antibodies in either sows or UCB samples (*p* > 0.05 for all comparisons) (Table 3).

## 4. Discussion

The 8.91% seropositivity for IgG antibodies against *N. caninum* in the serum of the sows was considerably higher than that reported in a previous investigation, where the prevalence was only 0.5% [17]. The significantly higher rate observed in the current study is likely a direct consequence of the targeted sampling strategy, as the selected population consisted exclusively of sows with litters with stillborn piglets. Consequently, this percentage should not be interpreted as representative of the general epidemiological situation of *N. caninum* exposure in Greek swine farms, but it reflects the potential contribution of *N. caninum* to reproductive disorders within specific affected herds. To our knowledge, this is the first study to report on IgM antibodies against *N. caninum* in swine, providing a new dimension to serological investigations.

Because *Toxoplasma gondii* and *N. caninum* are closely related coccidian parasites, they share several conserved antigens, including AMA1, protein disulfide isomerase (PDI), and heat-shock protein 70 (HSP70), and exhibit strong morphological similarity, factors that historically contributed to diagnostic confusion. These shared epitopes can lead to serological cross-reactivity, whereby antibodies generated against one parasite may bind to antigens of the other, potentially producing false-positive results in ELISA and indirect immunofluorescence assays (IFAs). Consequently, misinterpretation of serological data may occur, with *T. gondii* seropositivity occasionally reflecting underlying exposure to *N. caninum*, or vice versa [44]. Accurate diagnosis, therefore, requires either the use of highly specific assays or the application of elevated cut-off dilutions to minimize the impact of cross-reactive antibodies [34,44,45,46]. In this study, we acknowledge that the commercial kit used was developed for dogs and is not formally validated for swine, which represents a limitation. As the manufacturer does not specify the exact antigens incorporated on the slides and the kit documentation acknowledges the potential for cross-reactivity, the use of a higher cut-off dilution than the recommended threshold was deemed more appropriate to minimize false-positive reactions (1:160 instead of 1:50). Moreover, samples were considered as positive only if fluorescence was observed on the periphery of the tachyzoites excluding those with apical fluorescence which could be the result of cross-reaction with other Apicomplexa protozoa [43]. Nonetheless, these measures may not completely eliminate the possibility of cross-reactivity. Therefore, we recommend that future studies in this field incorporate parallel serological testing for both *T. gondii* and *N. caninum* to ensure species-specific responses are accurately identified.

A key finding of this study was the detection of IgG and/or IgM antibodies in 4.05% of UCB samples. Our study is based on serological findings alone, and a definitive diagnosis of active infection would ideally be confirmed by molecular techniques and/or histopathology on fetal tissues. This represents a limitation of our work. However, the porcine placenta has an epitheliochorial structure, which prevents the transfer of maternal immunoglobulins to the fetus [47]. As a result, any antibodies detected in fetal or umbilical cord circulation are of fetal origin and constitute direct evidence of in utero immune activation. This provides direct evidence of two biological events. First, the parasite successfully crossed the placental barrier to infect the fetus, which is consistent with previous findings [38,39], and second, the fetus developed its own active immune response to the infection.

The detection of IgM antibodies in UCB is particularly compelling, as IgM is the primary antibody in a nascent immune response. These findings gain even greater biological relevance when interpreted in light of the known development of the porcine immune system. Autoradiographic investigations into fetal immunoglobulin synthesis have shown that lymphocytes begin producing IgM, IgG, and IgA as early as the 38th day of gestation, corresponding to the first appearance of peripheral lymphocytes [48]. Results showed that during early gestation (days 38–58), few lymphocytes produced IgM, IgG, and IgA, but their numbers increased markedly after day 60, with IgM-producing cells remaining slightly predominant throughout [48]. Our findings suggest that UCB is a valuable biological sample for the diagnosis of vertically transmitted infections and for differentiating between maternal and fetal antibody profiles.

In the present study, IgG and/or IgM antibodies against *N. caninum* were detected in 4.05% of the UCB samples. This means that *N. caninum*-active tachyzoites transverse the placenta, an indication of active infection occurring at any stage of pregnancy, as a result of a primary oocyst-derived infection or of reactivation of a chronic latent infection [37]. It is further supported by the fact that IgM antibodies against *N. caninum* were detected in UCB without the detection of IgG or IgM antibodies in the mother’s serum. The presence of IgM antibodies in UCB in the absence of maternal IgG or IgM antibodies provides compelling evidence of an active de novo immune response in the fetus, indicating genuine intrauterine antibody production.

Sampling and antibody detection in sow and umbilical cord blood samples have been extensively used for monitoring porcine circovirus type 2 (PCV-2) [49] and porcine reproductive and respiratory syndrome (PRRS) to identify pathogen circulation within farms as well as efficacy of sow vaccination [27,50,51].

Our study confirms that this dual-sampling strategy is applicable to the study of congenital *N. caninum* infection as well, enhancing the understanding of infection dynamics in swine herds. By comparing antibody profiles between mothers and fetuses, it becomes possible to identify active transplacental transmission events, determine the timing of fetal infection, and assess the immunological status of both generations. Such approaches would contribute to a more accurate interpretation of serological results, especially in cases of reproductive failure, and could inform biosecurity strategies within breeding herds.

The immune changes that occur during gestation represent another important factor influencing the course of *N. caninum* infection. Pregnancy is characterized by a finely tuned state of immunological modulation, where certain responses are downregulated to ensure maternal–fetal tolerance, while others are enhanced to protect against pathogens. These shifts may alter the host’s susceptibility to infection, facilitate parasite reactivation, and exacerbate disease outcomes [52,53].

In the case of *N. caninum*, immunosuppression or immune deviation during gestation could enable tachyzoite reactivation and transplacental migration, leading to fetal infection or death, consistent with the pattern of abortions observed in affected farms [38,54].

Interestingly, in our study, the prevalence of *N. caninum*-positive samples in both sows and UCB was higher, though not to a statistically significant extent, in smaller-scale farms. This finding could be attributed to management-related factors such as lower biosecurity standards, less rigorous pest and dog control (dogs being the definitive hosts of *N. caninum*), suboptimal housing and hygiene conditions, or inconsistencies in feed storage and disposal practices. In contrast, larger farms often follow stricter biosecurity protocols, maintain controlled breeding programs, and apply more systematic monitoring schedules, all of which may limit exposure to the parasite. Although direct comparisons between small- and large-scale pig farms are scarce, available evidence suggests that smaller farms may face increased risk of *N. caninum* infection. In a study conducted in pig farms in Italy [23], higher seroprevalence was reported on farms with low sanitary scores—conditions typically associated with smaller, less-resourced operations. Similar patterns have been observed in other livestock species, where small herd size has been identified as a risk factor for *N. caninum* exposure. These differences highlight the importance of herd management practices in controlling *N. caninum* transmission and preventing reproductive losses.

From an epidemiological perspective, incorporating UCB sampling alongside maternal serology could offer a novel and cost-effective surveillance approach for the detection of congenital and subclinical infections. However, it is important to acknowledge that our study was exclusively focused on stillborn piglets. This design was chosen to allow the detection of a mature fetal immune response, as well as due to logistical challenges of a large, multi-farm study. Consequently, while our findings provide evidence of transplacental infection, they do not allow us to quantify the direct impact of *N. caninum* infection on overall abortion rates or other reproductive losses. Future longitudinal studies, following sows and their offspring through successive gestations, could provide valuable insights into the persistence, recrudescence, and vertical transmission rates of *N. caninum* under field conditions. Furthermore, correlating the immunological profiles of sows and fetuses with reproductive performance data such as conception rate, litter size, stillbirth frequency, and neonatal viability could elucidate the broader reproductive and economic impact of the parasite in swine production systems. Future epidemiological investigations would also benefit from employing multivariate statistical models to better understand the risk factors associated with *N. caninum* infection and farm management and sow health.

## 5. Conclusions

In conclusion, the results of the present study demonstrate that *N. caninum* infection in pigs is associated with detectable humoral responses in both sows and fetuses, reflecting active transplacental transmission and fetal immune activation. The concurrent detection of IgM and/or IgG antibodies in UCB demonstrates the immunocompetence of the porcine fetus and highlights the importance of using paired maternal and fetal samples for diagnostic and epidemiological purposes. This dual-sampling approach, successfully applied in PCV2 research, could significantly improve our understanding of *N. caninum* infection dynamics, guide preventive strategies, and enhance reproductive management in swine herds. Future research should adopt longitudinal designs and integrative methodologies combining serology, molecular detection, and reproductive outcome analysis to comprehensively assess the role of *N. caninum* in swine reproductive health.

## Figures and Tables

**Table 1:** Number of paired sow blood and umbilical cord blood (UCB) samples collected per farm of different farm sizes.

Farm Size ^1^	Farms	Paired Sow Blood and UCB Samples
50–100	10	42
101–250	16	95
251–500	10	80
>500	3	30
Total	39	247

^1^ Number of sows per farm.

**Table 2:** Number and percentage of positive samples for antibodies IgG and IgM against *N. caninum*, in total sow blood and total umbilical cord blood (UCB) samples.

Farm Size	Sows			UCB		
	IgG (+)	IgM (+)	IgG (+) and IgM (+)	IgG (+)	IgM (+)	IgG (+) and IgM (+)
50–100	7 (2.83%)	4 (1.62%)	1 (0.40%)	3 (1.21%)	1 (0.40%)	1 (0.40%)
101–250	8 (3.24%)	3 (1.21%)	1 (0.40%)	3 (1.21%)	1 (0.40%)	0 (0.00%)
251–500	6 (2.43%)	2 (0.81%)	0 (0.00%)	2 (0.81%)	0 (0.00%)	0 (0.00%)
>500	1 (0.40%)	0 (0.00%)	0 (0.00%)	0 (0.00%)	0 (0.00%)	0 (0.00%)
Total	22 (8.91%)	9 (3.64%)	2 (0.81%)	8 (3.24%)	2 (0.81%)	1 (0.40%)

**Table 3:** Positive samples for IgG and/or IgM antibodies against *N. caninum*. in sows and umbilical cords per farm size.

Farm Size	N	Sows						Umbilical Cord					
		IgG-Positive		IgM-Positive		IgG-IgM Positive		IgG-Positive		IgM-Positive		IgG-IgM Positive	
		N (%)	*p*-Value	N (%)	*p*-Value	N (%)	*p*-Value	N (%)	*p*-Value	N (%)	*p*-Value	N (%)	*p*-Value
50–100	42	7 (16.67)	0.213	4 (9.52)	0.19	1 (2.38)	0.603	3 (7.14)	0.457	1 (2.38)	0.603	1 (2.38)	0.292
101–250	95	8 (8.42)		3 (3.16)		1 (1.05)		3 (3.16)		1 (1.05)		0 (0.00)	
251–500	80	6 (7.50)		2 (2.50)		0 (0.00)		2 (2.50)		0 (0.00)		0 (0.00)	
>500	30	1 (3.33)		0 (0.00)		0 (0.00)		0 (0.00)		0 (0.00)		0 (0.00)	
Total	247	22 (8.91)		9 (3.64)		2 (0.81)		8 (3.24)		2 (0.81)		1 (0.41)	

## Data Availability

The data presented in this study are available on request from the corresponding author. The data are not publicly available due to further processing for other studies.
